# The uterine expression of *SEC63 *gene is up-regulated at implantation sites in association with the decidualization during the early pregnancy in mice

**DOI:** 10.1186/1477-7827-7-12

**Published:** 2009-02-11

**Authors:** Ren-wei Su, Zhao-gui Sun, Yue-chao Zhao, Qiu-ju Chen, Zeng-ming Yang, Run-sheng Li, Jian Wang

**Affiliations:** 1 School of Life Science, Xiamen University, Xiamen 361005, PR China; 2 Key Laboratory of Contraceptives and Devices of National Population & Family Planning Committee, Shanghai Institute of Planned Parenthood Research, Shanghai 200032, PR China

## Abstract

**Background:**

Sec63 is a key component of the protein translocation machinery in the mammalian endoplasmic reticulum (ER), and involved in the post-translation processing of secretory proteins. The aim of this study was to determine the expression pattern of SEC63 gene in mouse uterus during the early pregnancy.

**Methods:**

Real-time quantitative PCR and Western blot analyses were used to evaluate the alteration in levels of uterine *SEC63 *gene expression during the peri-implantation period in mice. Further, both in situ hybridization and immunohistochemical analyses were performed to examine the spatial localization of SEC63 gene expression in mouse uterine tissues. The presence of Sec63 protein in human uterine tissue was also detected by immunohistochemical analysis. Statistical analysis was carried out using Tukey test.

**Results:**

Uterine SEC63 gene expression was up-regulated and predominantly localized in mouse decidual cells during days 5–8 of pregnancy. More interestingly, Sec63 protein was also detected in human decidua of 10-week pregnancy, whereas was not observed in human endometrial tissues both at proliferative and secretory phases of menstrual cycle.

**Conclusion:**

The pattern of SEC63 gene expression is consistent with a possible role for SEC63 in decidualization.

## Background

Embryo implantation is a critical step in pregnancy and currently considered the most relevant limiting factor for successful pregnancy [1,2]. Successful implantation depends on the synchronized development of a normal embryo to the blastocyst stage, and the maternal uterus from a non-receptive to a receptive state, as well as the establishment of the active interactions between maternal and embryonic tissues [3,4]. This exquisite coordination involves the regulated production of hormonal and non-hormonal molecules by embryonic and maternal tissues [5,6]. A large number of non-hormonal factors have been identified to be involved in this process, and some of them have been extensively investigated and regarded as the endometrial receptivity markers [4,7]. However, the exact molecular interactions between the implanting embryo and the maternal uterus are still not clear. To identify novel genes that could be crucial for embryo implantation and to explore their biological roles in implantation would undoubtedly accelerate a better insight into the molecular mechanism underlying embryo implantation.

In order to search for the novel molecules that are highly expressed at the implantation sites, we have successfully applied the CLONTECH PCR-select cDNA subtraction technique to screen specifically up-regulated genes in the mouse uterus around the time of implantation [8,9]. One of the genes screened out from the subtracted cDNA library was *SEC63 *gene that encodes Sec63 protein (Sec63p). Sec63p is involved in the post-translational processing of secretory proteins [10], including the folding and quality control of secretory proteins [11,12], as a component of the protein translocation machinery in the endoplasmic reticulum (ER) of eukaryotic cells [13,14]. *SEC63 *expression was originally found in Saccharomyces cerevisiae [15]. The mammalian *SEC63 *cDNA and Sec63p were also identified subsequently [16]. Mammalian Sec63p consists of 760 amino acids, sharing 53% homology and 25.6% identity with the yeast Sec63p [16]. As an ER integral membrane protein of the Hsp40 family [17,18], Sec63p could facilitate protein translocation into the ER. The C-terminal conserved Brr2-like domain of Sec63p, that could be phosphorylated by the protein kinase CK2, is essential for its function [18,19]. Sec63p is required for post-translational translocation of invertase, carboxypeptidase Y (CPY) and dipeptidyl-aminopeptidase B (DPAP B) in yeast [15,20,21]. In mammals, Sec63p is a prime candidate for co-chaperone of IgG heavy chain-binding protein (BiP/Kar2p) in protein transport [22]. However, the exact secretory protein species of Sec63p-dependent secretion in mammals is still unclear. Because mammalian uteri synthesize secretory proteins essential for survival and development of the embryo and fetus during pregnancy [23], we hypothesize that Sec63p may also be involved in the process of embryo implantation. Thus, the present study was undertaken to examine the pattern of SEC63 gene expression in the uterus during the peri-implantation period in mice by *in situ *hybridization and immunohistochemistry.

## Methods

### Animals and tissue preparation

Adult ICR mice aged 6–8 weeks were obtained from the SIPPR/BK Laboratory Animal Company (Shanghai, China). All of the mice were caged at controlled temperature (approximately 22°C) under a 14 h light: 10 h dark photoperiod. All the experiments were in full compliance with standard laboratory animal care protocols (NTC-SOP-ADM-ANM-003, -014, -038, -039 and -040) approved by the Institutional Animal Care Committee of Shanghai Institute of Planned Parenthood Research.

Adult females were mated with fertile males of the same strain to achieve pregnancy (day1 = day of vaginal plug). Pregnancy was confirmed on days 1–4 by recovering embryos from the reproductive tracts. The implantation sites on days 5–6 were identified by intravenous injection of Chicago blue dye solution (1% in saline; 0.1 ml per mouse; Sigma, St Louis, MO). The entire uteri of non-pregnant and pregnant mice on days 1–4 pregnancy were respectively collected immediately after the mice were sacrificed by cervical dislocation. For days 5–8 of pregnancy, uterine tissues at implantation sites and inter-implantation sites were separately collected. Embryos were collected by flushing the uterus with PBS at 07:00–08:00 on day 5 of pregnancy.

Pseudopregnant mice were obtained by mating females with vasectomized males, and the entire uteri of pseudopregnant mice on days 1–5 were collected. Implantation was delayed by ovariectomizing pregnant mice at 08:30–09:00 h on day 4 of pregnancy and administering progesterone (1 mg per mouse, Sigma) during days 5–7. Delayed implantation was subsequently initiated by administering estradiol-17β (25 ng per mouse, Sigma) on day 7. The uterine tissues were respectively collected at 24 h after estrogen or last progesterone treatment. Delayed implantation was confirmed by flushing the blastocysts from uterus, and the activation of delayed implantation was identified by intravenous injection of Chicago blue dye solution [8,9].

Ovary and non-reproductive tissues including brain, lung, heart, liver, spleen, kidney, muscle, small intestine, skin were also collected from the day 5 pregnant mouse. Male reproductive tissues, testis and epididymis, were collected from adult male mice. All tissues collected were stored at -80°C until further analysis.

The sections of human decidual and oviductal tissues were kindly provided by the International Peace Maternity and Child Healthcare Hospital, the China Welfare Institute, Shanghai, China. Ethical approvals were granted both from Shanghai Institute of Planned Parenthood Research, and the Department of Obstetrics and Gynecology, Shanghai Jiao Tong University. All subjects signed an informed consent before the collection of decidual and oviductal tissues.

### Reverse transcription (RT)-PCR

Different tissues, including brain, lung, heart, liver, spleen, kidney, muscle, small intestine, skin, ovary, uterus, testis and epididymis, were individually collected from 3 mice and pooled. Total RNA was then respectively extracted from each tissue pool using Trizol reagent (Invitrogen Life Technologies Ltd, Renfrew, Strathclyde, UK), and the RNA quality was evaluated by the ratio of optical density (OD_260_: OD_280_). Only these RNA samples with a ratio > 1.8 were used for PCR. Each RNA sample (2 μg) was treated by RNase-free DNase I (TakaRa Biotechnology Co.) at 37°C for 30 min. RNA Reverse transcription (RT) was performed at 42°C for 1 h with the poly(dT) primer in 50 μl reaction mixture using Superscript MII reverse transcriptase (Gibco BRL, Invitrogen Corporation, Carlsbad, CA) according to the manufacturer's instructions. Then, 2 μl RT reaction mixture were used as the template for PCR amplification at the cycling conditions: 94°C 20 sec, 57°C 20 sec, 72°C 30 sec for 35 cycles. The primers used to detect the targeted and housekeeping control mRNAs were as follows: *SEC63*, 5'-AAGAATGAACCTCCACTCACC-3' (sense) and 5'-GTCCTCTTCAATATGAGGGAGC-3' (antisense) (318 bp); *GAPDH*, 5'-GTCGGTGTGAACGGATTT-3' (sense) and 5'-ACTCCACGACATACTCAGC-3' (antisense) (276 bp). PCR products were separated on 2% agarose gel and stained with ethidium bromide (EB), and the signal was photographed by BioSens Gel Imaging System (Shanghai Bio-Tech Co., Ltd., Shanghai, China). The RT-PCR reaction conditions were optimized, specific product was obtained and verified by sequencing.

### Real-time quantitative PCR analysis

Uterine expression levels of *SEC63 *mRNA on days 4, 5 and 7, as well as the inter-implantation sites and implantation sites of day 7 pregnant mice, were relatively determined by real-time quantitative PCR analysis using the level of β-*Actin *mRNA as the reference. Uterine tissues were collected from 3 mice at each time-point and pooled. Total RNA samples were isolated and RT reactions were performed as above described. The primers were as follows: *SEC63*, 5'-TGACAAGGGCAGTGATTCTG-3' (sense) and 5'-GCCACCACC ACTCTTGTTTT-3' (antisense) (248 bp); β-*Actin*, 5'-AGCCATGTACGTAGCCATCC-3'(sense) and 5' CTCTCAGCTGTGGTGGTGAA-3' (antisense) (228 bp). All amplifications were designed to span over introns to rule out the genomic DNA contamination, and the PCR products were authenticated by sequencing. The PCR reaction mixture (25 μl) contained 12.5 μl SYBR green I reaction (QPK-201, Toyobo Co., Ltd., Osaka, Japan). The reactions were performed on the Peltier Thermal Cycler (Bio-Rad Laboratories, Hercules, CA) at the conditions: 94°C 15 sec, 55°C 30 sec, 72°C 45 sec for 46 cycles. The RT-PCR conditions were optimized and the melting curve presents a single amplified band. The Ct data were imported into Microsoft Excel, the relative value of *Sec63 *mRNA copies was obtained according to 2^-ΔΔCt ^method [24]. Triplicate PCR reactions were run simultaneously, and the mean of measured values was used to get the ratio between *SEC63 *mRNA and β-*Actin *mRNA, to represent the relative expression level of *SEC63 *mRNA. The data from 3 independent experiments (triplicate of the pooled data, from a total of 9 mice) were taken for statistical treatments.

### Western blot analysis

Following a described protocol [25], samples of total proteins were isolated respectively from the mouse uterine tissues on days 1 to 7 of pregnancy, separated on the sodium dodecyl sulfate (SDS) polyacrylamide gel electrophoresis (100 μg proteins per well), and transferred to a nitrocellulose membrane (Hybond™-C, Amersham Biosciences, Piscataway, NJ). The membrane was washed several times with 10% skim milk powder in TBS with 0.1% Tween-20 (Sigma, St. Louis, MO, USA) and incubated with the goat anti-sera specific for both mouse and human Sec63p (1:50, Santa Cruz Biotechnology Inc, Santa Cruz, CA) in blocking solution (7 mM Na_2_HPO_4_, 2.5 mM NaH_2_PO_4_, 140 mM NaCl, 4.5% skim milk powder) for 2 h. Then the membrane was washed and incubated with alkaline phosphatase-labeled secondary antibodies (ZYMED, S. San Francisco, CA) in blocking solution for 2 hrs at room temperature. After further washing, the signal was visualized with BCIP/NBT (ZYMED) in 0.1 M Tris·HCl (pH 9.5) and 4 mM MgCl_2_. Comparable protein loading per lane was confirmed by using β-actin as the reference. The rabbit anti-β-actin antibody (Abcam, Cambrige, UK) was used as the primary antibody, and the AP-labeled donkey antibody against rabbit IgG (ZYMED) was used as the secondary antibody. Normal goat IgG was used as the primary antibody in parallel to check the immunological specificity.

### *In situ *hybridization

A 450 bp (C^65 ^– G^514^) *SEC63 *cDNA fragment (Accession no. AY024346) was obtained by restriction digestion of the plasmid pMD18-T/SEC63 using Nco I and Nde I. The plasmid contained the full length 2558 bp *SEC63 *cDNA, kindly provided by Professor Cheng-quan Liu, Shanghai Institute of Planned Parenthood Research. The *SEC63 *cDNA fragment (*SEC63f*) was subcloned into the pGEM-T at Nco I and Nde I restruction sites. Then the recombinant plasmid pGEM-T/SEC63f was linearized for labeling. Digoxigenin (DIG)-labeled antisense or sense cRNA probes were transcribed in vitro using a DIG RNA labeling kit (Roche Diagnostics Corp., Indianapolis, IN).

Uteri were cut into 4–6 mm pieces and flash frozen in liquid nitrogen. Frozen sections (10 μm) were mounted on 3-aminopropyltriethoxy-silane-coated (Sigma, St Louis, MO) slides and fixed in 4% (w/v) paraformaldehyde solution in PBS. The sections were washed twice in PBS, placed in 1% (v/v) Trition-X100 for 20 min and washed again in PBS three times. After the pre-hybridization in the solution of 50% formamide and 5 × SSC (1 × SSC is 0.15 M sodium chloride, 0.015 M sodium citrate) at room temperature for 15 min, the sections were hybridized in the hybridization buffer (5 × SSC, 50% formamide, 0.02% BSA, 250 μg yeast tRNA ml^-1^, 10% (w/v) dextran sulphate, 1 μg ml^-1 ^denatured DIG-labelled antisense or sense RNA probe for mouse *SEC63 *mRNA) at 55°C for 16 h. After hybridization, the sections were washed in 50% formamide/5 × SSC at 55°C for 15 min, 50% formamide/2 × SSC at 55°C for 30 min, 50% formamide/0.2 × SSC at 55°C twice for 30 min each, 0.2 × SSC at room temperature for 5 min and buffer I (100 mM Tris-HCl, pH 7.5, 150 mM NaCl) for 5 min. After non-specific binding was blocked in 1% block mix (Boehringer Mannheim) in buffer I for 1 h, sections were incubated in sheep anti-DIG antibody conjugated with alkaline phosphatase (1:5000, Boehringer Mannheim) in 1% block mix at 4°C overnight. The signal was visualized with 0.4 mM BCIP/NBT in the buffer III (100 mM Tris-HCl, pH 9.5, 100 mM NaCl and 50 mM MgCl_2_). Endogenous alkaline phosphatase activity was inhibited with 2 mM levamisole (Sigma). All of the sections were counterstained with 1% (w/v) methyl green, in 0.12 M glacial acetic acid and 0.08 M sodium acetate, for 30 min. The positive signal was visualized as a dark-brown color.

### Immunohistochemistry

#### Mouse tissues

Immediately after mice were killed by cervical dislocation, mouse uterine tissues were cut into 4–6 mm long pieces, fixed in Bouin's solutions for 24 h, dehydrated and embedded in paraffin (Beijing Chemical Co., Beijing, China). Sections (7 μm) were cut, deparaffinized and rehydrated. Nonspecific binding was blocked in 10% normal rabbit serum in PBS for 1 h at 37°C. Sections were incubated with goat anti-sera specific for both mouse and human Sec63 proteins (1:100, Santa Cruz Biotechnology Inc) overnight at 4°C. After washing in PBS three times, sections were incubated with biotinylated rabbit anti-goat IgG (1:200, Vector Laboratories, Burlingame, CA, USA), followed by an avidin-alkaline phosphatase (ALP) complex and Vector red using the Vectastain ABC-AP kit (Vector Laboratories). Vector red was visualized as a red color. Endogenous ALP activity was blocked by incubation with 2 mM levamisole (Sigma). Sections were counterstained with hematoxylin (Sigma) and mounted. Negative controls were performed by adding blocking peptides (8μg/μg goat anti-Sec63 antibody) into the primary antibody solution prior to addition of the solution to tissue sections.

#### Human tissues

The serial 6 μm-thick sections of human decidual and oviductal tissues, mounted on Poly-L-Lysine (Sigma) coated glass slides, were incubated with 10% normal donkey serum in PBST (PBS, pH7.4, containing 0.2% Tween-20) for 1 h to block the nonspecific binding. Sec63 antigens were recognized by goat anti-sera specific for both mouse and human Sec63p (1:50, Santa Cruz Biotechnology Inc) overnight at 4°C. After washing for 3 times in PBST, the sections were incubated with biotin-conjugated donkey anti-rabbit IgG (1:200, Abcam Plc, Cambridge, UK) for 1 hour at 37°C. After washing, strepavidin-HRP (Proteintech Group Inc, Chicago, IL) was added to bind with biotin. DAB was used as substrate for the peroxidase reaction. Sections were counterstained with hematoxylin, dehydrated and mounted. As a control, the blocking peptides neutralized antisera (8μg/μg goat anti-Sec63 antibody) was used at the same dilution.

### Statistical analysis

The data were processed and the bar graphs were obtained by SPSS13.0 (SPSS Inc., Chicago, IL). All the numerical results are expressed as mean ± SE (standard error of mean), and variances were analyzed for statistical significance using one-way analysis of variance with p < 0.05. The multiple comparisons were conducted with the Tukey Method to analyze the differences of uterine SEC63 mRNA levels at days 4, 5 and 7 of pregnancy in real-time quantitative PCR analysis.

## Results

### Tissue distribution of *SEC63 *mRNA

**Figure 1:**
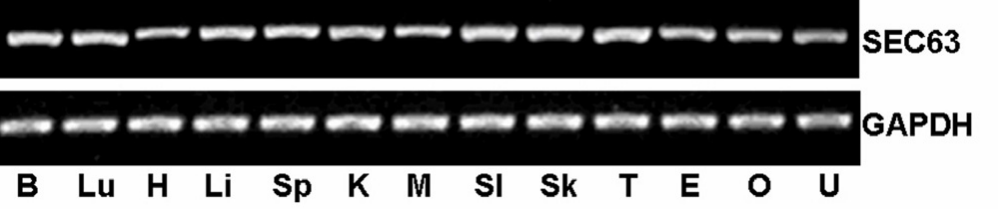
**The tissue distribution of *Sec63 *mRNA in the mouse**. The tissue distribution of *Sec63 *mRNA in the mouse was analyzed by RT-PCR by using *Gapdh *mRNA as the control. *Sec63 *mRNA was detected in all of the tested tissues. (B: brain; Lu: lung; H: heart; Li: liver; Sp: spleen; K: kidney; M: muscle; SI: small intestine; Sk: skin; T: testis; E: epididymis; O: ovary; U: uterus.)

Multi-tissue RT-PCR analysis was performed to observe the tissue distribution of the *SEC63 *gene expression in different mouse tissues using *GAPDH *as the housekeeping control. The results showed that *SEC63 *mRNA was widely expressed in all of the tissues tested (Fig. 1).

### Uterine expression level of *SEC63 *mRNA during peri-implantation period

**Figure 2:**
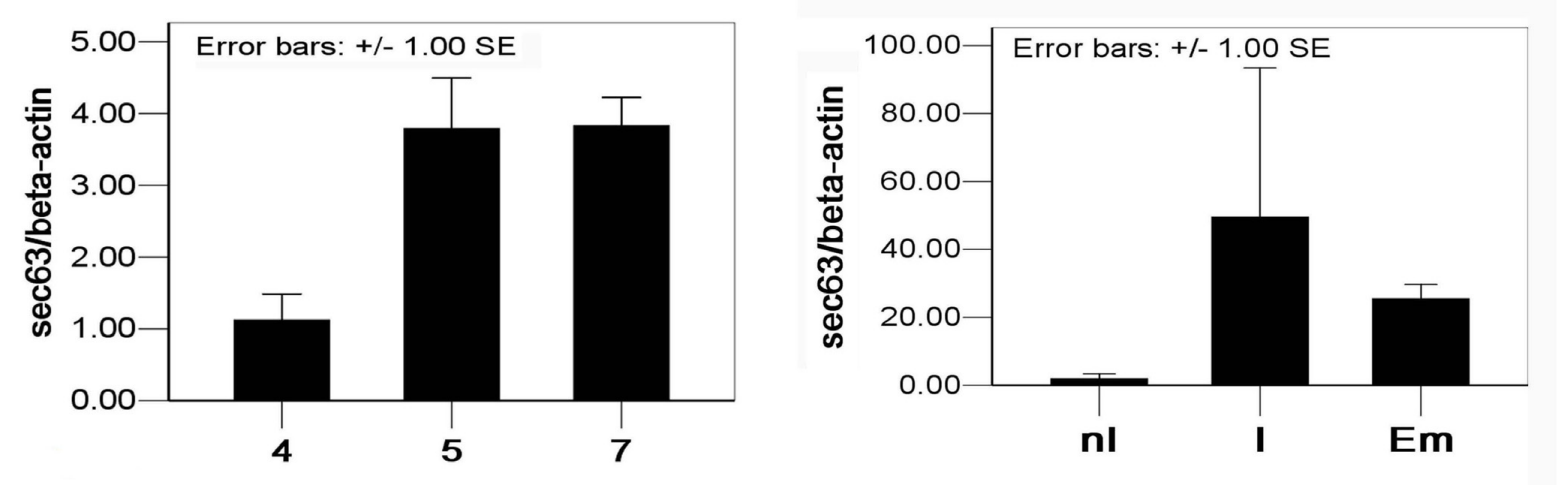
**Uterine expression level of *Sec63 *mRNA during the early pregnancy**. Real-time quantitative PCR analysis of the expression level of *Sec63 *mRNA in mouse uterine tissues of day 4 (4), day 5 (5) and day 7 (7) pregnancy (panel A), as well as the inter-implantation sites (nI), implantation sites (I) and embryo (Em) of day 7 pregnancy (panel B), by using the β-*Actin *as the housekeeping gene. In panel A and panel B, the data were normalized relative to that of day 4 and nI (reference groups) respectively.

The uterine expression levels of *SEC63 *mRNA of days 4, 5 and 7 were determined by real-time quantitative PCR analysis (See additional file 1 for Melting curve of real-time quantitative PCR analysis for *Sec63 *mRNA). Compared to that of day 4 pregnant mouse, the measured values of *SEC63 *mRNA in the mouse uterus on days 5 and 7 of pregnancy were significantly increased (Fig. 2A and Table 1). Furthermore, the expression level of *SEC63 *mRNA was up-regulated in tendency at the implantation sites compared to the inter-implantation sites on day 7 pregnancy (Fig. 2B), despite of the great variance possibly resulting from the dissection operation of the implantation site and the non-implantation site.

**Table 1:** Uterine *Sec63 *mRNA levels of days 4, 5 and 7 pregnant mice determined by real-time quantitative PCR

					**95% Confidence Interval**	
					
**(I) Sample**	**(J) Sample**	**Mean Difference (I-J)**	**Std. Error**	**Sig.**	**Lower Bound**	**Upper Bound**
Day 4	Day 5	-2.66558*	.72340	.024*	-4.8852	-.4460
	Day 7	-2.70786 *	.72340	.022*	-4.9275	-.4883

Day 5	Day 4	2.66558 *	.72340	.024*	.4460	4.8852
	Day 7	-.04228	.72340	.998	-2.2619	2.1773

Day 7	Day 4	2.70786*	.72340	.022*	.4883	4.9275
	Day 5	.04228	.72340	.998	-2.1773	2.2619

(*: the mean difference is significant at the .05 level)

### Production of Sec63p in the mouse uterus during early pregnancy

**Figure 3:**
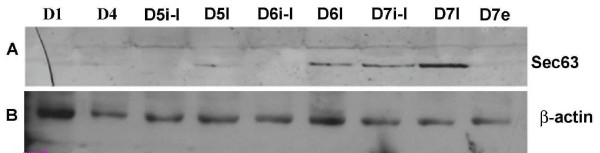
**Uterine expression level of SEC63 protein during the early pregnancy**. Western blot analysis of SEC63 expressed in mouse uterine tissues during the early pregnancy using the goat anti-sera specific for both mouse and human Sec63p (1:50) as the primary antibody. Total protein (100 μg) was exacted from uteri of day 1 (D1) and day 4 (D4) pregnant mice, and from inter-implantation sites (i-I) and implantation sites (I) on day 5 (D5) and day 6 (D6). On day 7 (D7), embryos (e) were also separated from the implantation sites. Panel A shows the SEC63 signal detected by goat anti-sera specific for both mouse and human Sec63p. Panel B shows the equal of loading samples stained by the rabbit anti-β-actin antibody.

The mouse Sec63p produced in the uterus during early pregnancy was detected by Western blot. Sec63p was detectable in the implantation sites on day 5 of pregnancy, and increased from day 6 to day 7 of pregnancy. In addition, the Sec63 gene expression was up-regulated in the implantation sites relative to that in the inter-implantation sites on days 5–7 of pregnancy (Fig. 3).

### *In situ *hybridization of *SEC63 *mRNA in the mouse uterus during early pregnancy

**Figure 4:**
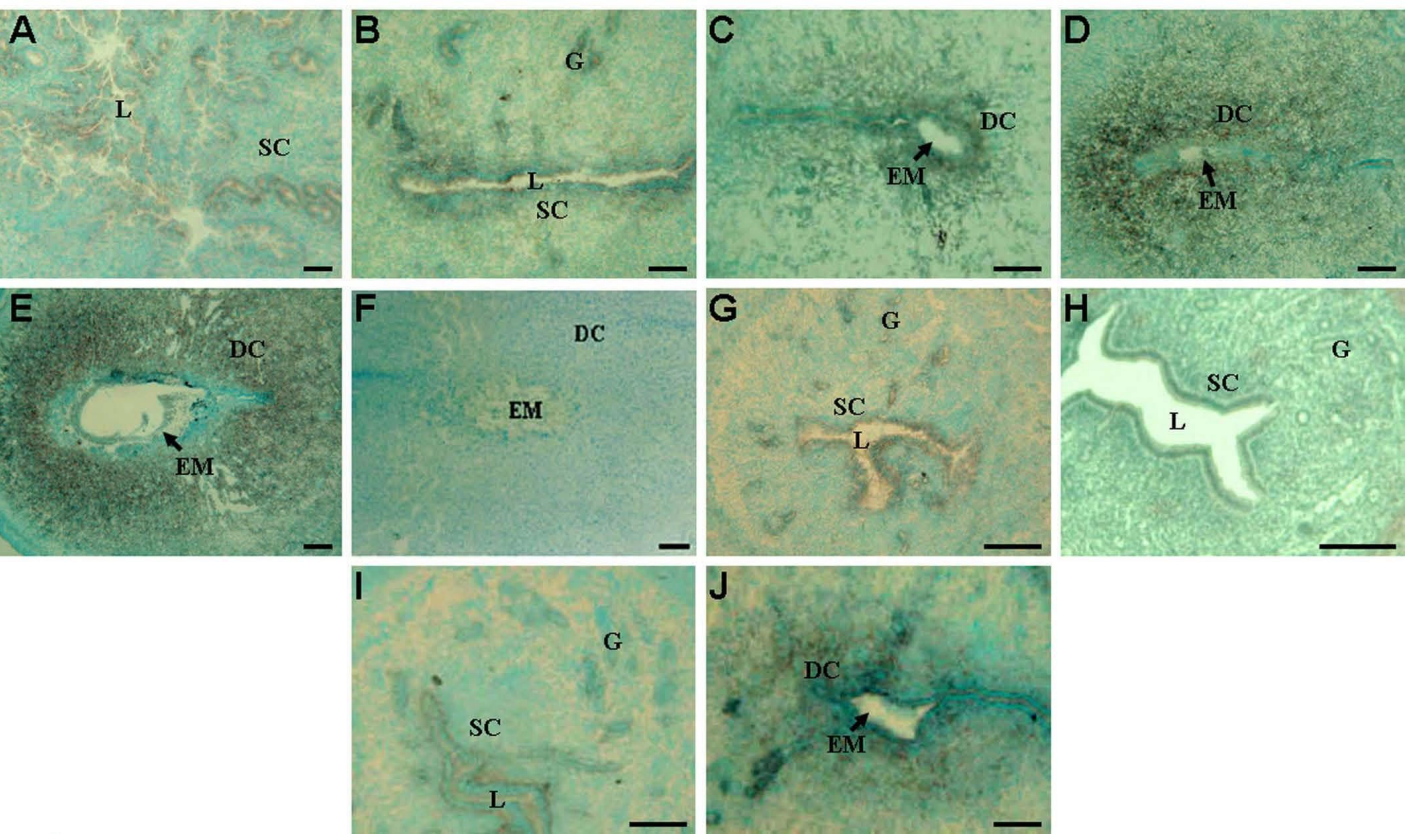
**Determination of uterine *Sec63 *mRNA in the mouse by *in situ *hybridization analysis**. *In situ *hybridization of *Sec63 *mRNA was performed using digoxigenin-labeled anti-sense probes generated against *Sec63 *cDNA in the mouse uterus on day 1(A) and day 4 (B) of pregnancy; at the implantation site of day 5(C), day 6 (D) and day 8 (E); at the inter-implantation site of day 6 (H); in the uterus of day 5 pseudopregnant mouse (G); delayed implantation (I) before and (J) after activation. (F) is the same as (E), but sense sequence was used for substitution as control. L: lumen of uterus; G: gland; SC: stromal cells; DC: decidual cells; EM: embryo. Scale bars represents 50 μm.

The expression of *SEC63 *mRNA in the uterus during the peri-implantation period was examined by *in situ *hybridization. Only weak signals were detected in the epithelial cells of uterine lumen and glands on day 1 (Fig. 4A), days 2–3 (data not shown) and day 4 (Fig. 4B). However, from days 5 to 8 of pregnancy, more intense *SEC63 *mRNA expression was predominantly observed in decidual cells around the implanted embryo at the implantation site (Fig. 4C, D, E), whereas expression at the inter-implantation site was dramatically reduced (Fig. 4H). In pseudopregnant mice, weak Sec63 expression were observed in the epithelial cells of uterine lumen and glands on day 5 (Fig 4G). Under delayed implantation, there was no intense detection of Sec63 mRNA in the uterus (Fig 4I), whereas intense Sec63 expression was mainly localized in the decidualization area at the implantation site after the activation of embryo implantation (Fig 4J).

### Uterine expression pattern of Sec63p during early pregnancy in mice

**Figure 5:**
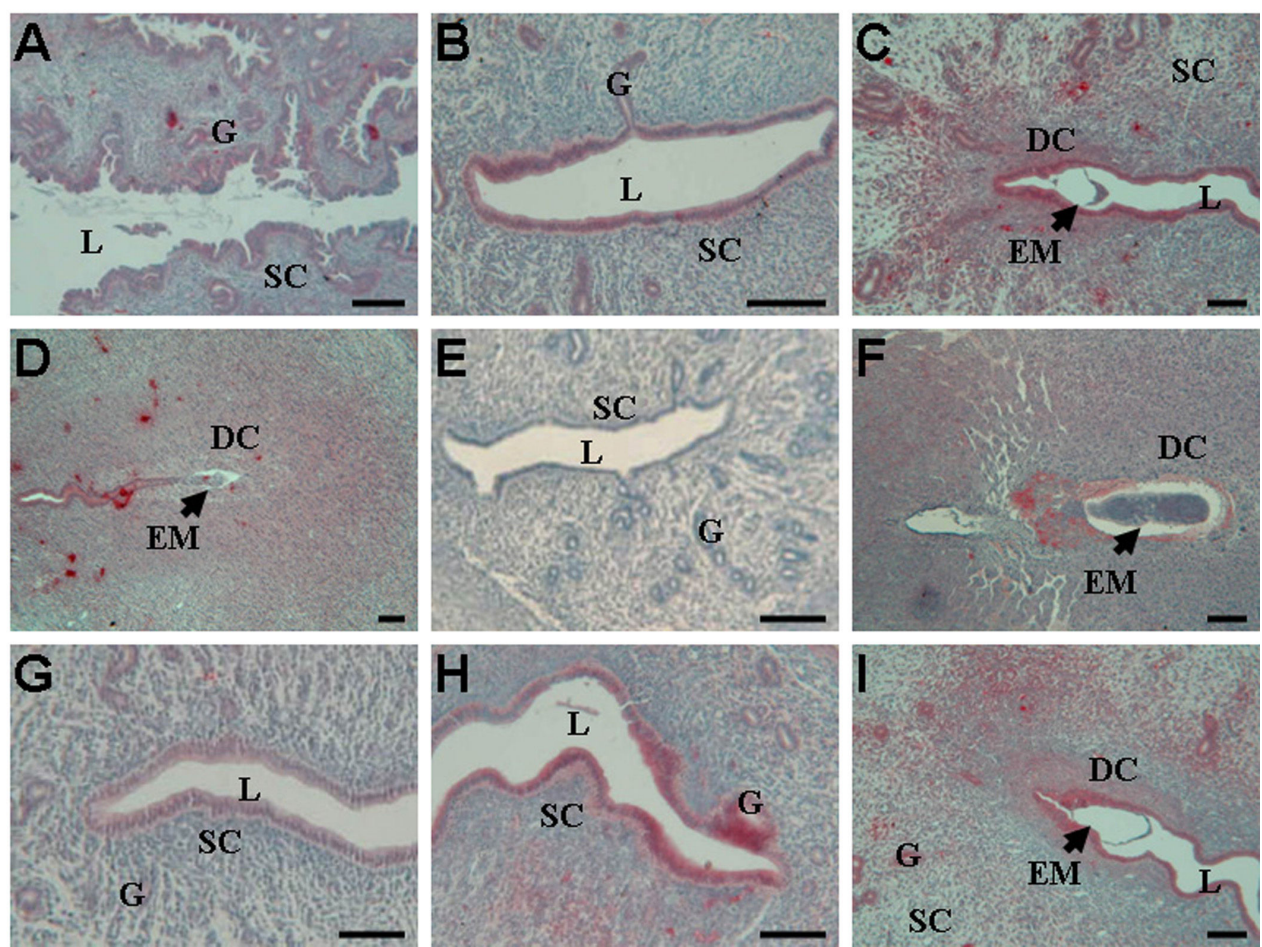
**Examination of uterine SEC63 protein in the mouse by Immunohistochemical staining**. SEC63p immunohistochemical staining of mouse uterine tissues was performed by using goat anti-sera specific for both mouse and human Sec63p as the primary antibody. The uterine sections were stained with Sec63 for day 1 (A) and day 4 (B) pregnant uterus; the implantation site of day 5 (C), day 6 (D) and day 8 (F); day 5 pseudopregnant uterus (G); and delayed implantation before (H) and 24 hr after activation (I). Panel E is the negative control for panel B, in which normal goat serum is substituted for the primary antibody. L: lumen of uterus; G: gland; SC: stromal cells; DC: decidual cells; EM: embryo. Scale bars represents 10 μm.

Immunohistochemistry was conducted to determine whether Sec63p was present in the mouse uterus. Only weak immunostaining of Sec63p was detected in the epithelial cells of uterine lumen and glands on day 1 (Fig. 5A), days 2–3 (data not shown) and day 4 (Fig. 5B). Strong immunostaining of Sec63p was observed in the epithelial cells and decidualized area around the implanted embryo at the implantation site on days 5, 6 and 8 of pregnancy (Fig. 5C, D, F), whereas the staining intensity at the inter-implantation site was sharply decreased (data not shown). Furthermore, intense staining of Sec63p was also discerned in the ectoplacental cone at the implantation site on day 8 (Fig. 5F). In pseudopregnant mice, faint staining of Sec63p was observed in the epithelial cells of uterine lumen and glands on day5 (Fig 5G). Similarly, some weak Sec63p staining was detected in the epithelial cells of uterine lumen and glands under delayed implantation (Fig. 5H), but more intense Sec63p staining was localized in the decidualized area, as well as in the epithelial cells of uterine lumen and glands, at the implantation site after the activation of embryo implantation (Fig. 5I).

### Expression of Sec63p in human decidual and oviductal tissues

**Figure 6:**
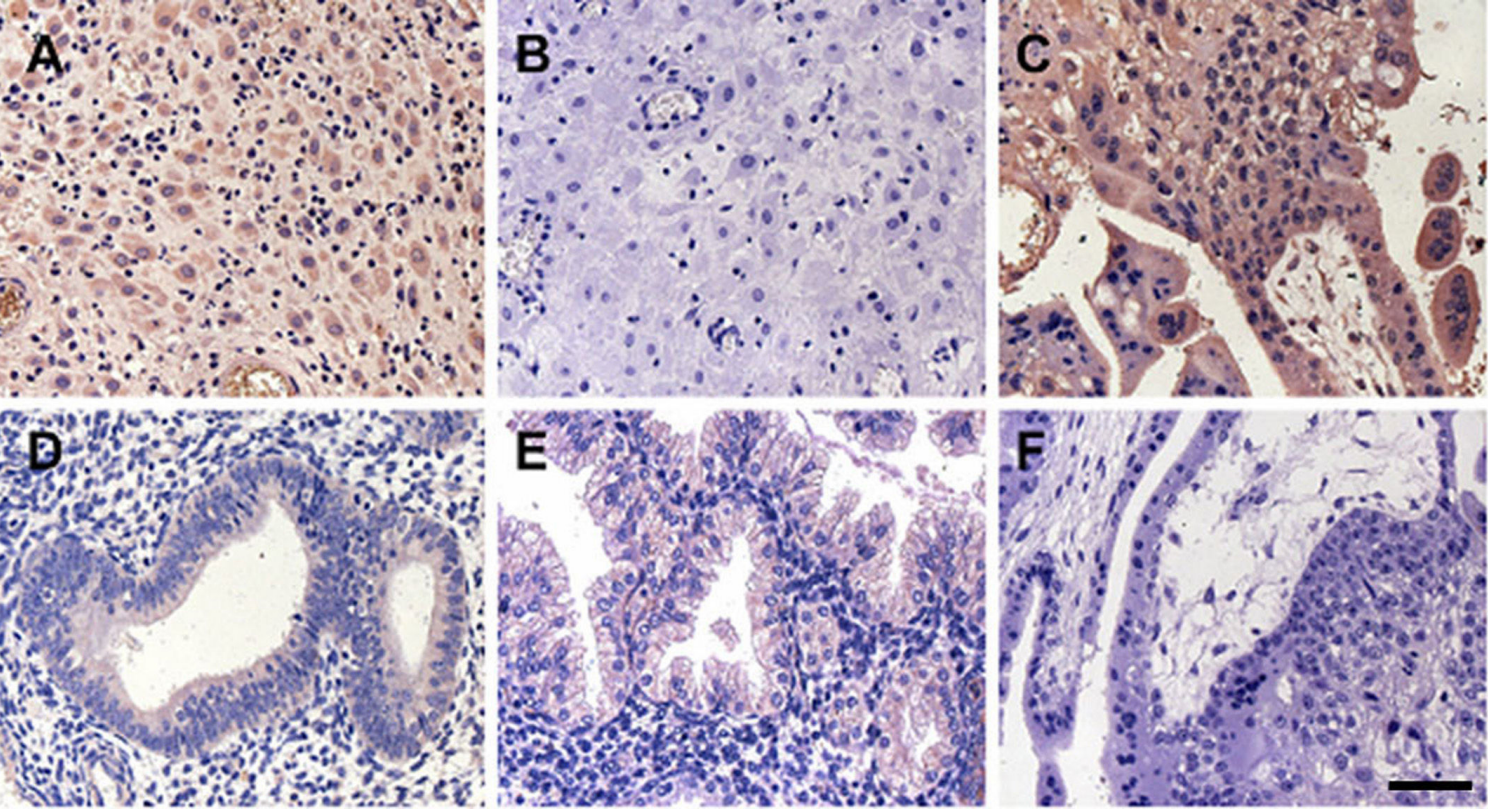
**The present of SEC63 in human tissues detected by IHC analysis**. Immunohistochemical staining for SEC63 in human decidua of 10-week pregnancy (A), human oviduct of 10-week ectopic pregnancy (C), human endometrium at proliferative phase (D) and human endometrium at secretory phase (E) by using goat anti-sera specific for both mouse and human Sec63p as the primary antibody. Panel B and F are the negative controls respectively for panel A and C, in which normal goat serum is substituted for the primary antibody. Scale bars represents 50 μm.

The production of Sec63p in the human uterine and oviductal tissues was also determined by immunohistochemistry using the goat anti-sera specific for both mouse and human Sec63p. Intense Sec63p staining signals were observed in decidualized cells of uterine and oviductal tissues (Fig. 6A, C); whereas the staining was faintly detected in the gland epithelial cells of human endometrium at proliferative (Fig. 6D) and secretory phase of the menstrual cycle (Fig. 6E).

## Discussion

Decidualization, characterized by growth and differentiation of endometrial stromal cells, is a critical event occurred during embryo implantation. In the present study, we identified the predominant expression of *SEC63 *mRNA and Sec63p in the decidualized area of uterine tissues during early pregnancy in mice. More interestingly, an increased expression of Sec63p was also observed in human decidualized uterine and oviductal tissues.

During the pregnancy, the fetomaternal interface, consisting of the maternal decidua and the invading fetal trophoblast, critically regulates placental function and the growth and development of the conceptus. The maternal decidua is differentiated from the endometrial tissues during decidualization, a postovulatory process of endometrial remodeling in preparation for pregnancy [26,27]. In most species studied, during the embryo implantation process, an increase of endometrial vascular permeability in areas adjacent to the blastocysts is the earliest macroscopically identifiable change, and, this is followed in species with invasive implantation by decidualization [28,29]. Meanwhile, molecules produced by endometrial epithelium, including cytokines, chemokines, growth factors, and proteinases, could be secreted into the uterine lumen to affect blastocyst development, migration, and attachment, or to basally facilitate the differentiation of the underlying stromal cells to produce decidual cells; and these decidual cells, which subsequently form a major component of the decidua of pregnancy, also produce a set of secreted proteins, such as insulin-like growth factor binding protein-1 (IGFBP1) [30] and placental protein 14 (PP14) [31], to drive the decidualization process [2,32]. Therefore, the process of decidualization is accompanied by the frequent production and secretion of a number of secretary proteins in endometrial tissues which fills the intrauterine cavity and glandular lumen [33].

The targeting and transport of precursors of secretary proteins into the endoplasmic reticulum (ER) is essentially required for the biosynthesis of most secretory proteins [10,34], and the protein translocation channel in the ER membrane mediates such a transport [11]. Specific protein complexes that recognize the signal sequence of these precursors are involved in their translocation to the membrane of the ER [10]. However, different translocation substrates appear to have different requirements for components of the cellular targeting and translocation machinery [35]. The major components of translocation machinery are signal recognition particle (SRP) and secretory (Sec) complex [36]. The later consists of Sec61p, Sec62p, Sec63p, Sec71p and Sec72p[10,18]. However, being different from the other Sec complex components, Sec63 is also involved in the SRP-dependent transport pathway [13]. Dysfunction of the SEC63 gene is associated with defects in protein translocation into the ER[37] and results in human diseases including various cancers [29] and the polycystic liver disease (PCLD) [12,38].

The precise bio-functions of Sec63p in mammals, especially the role it plays in reproduction, are still unclear. The group of secretary proteins of which precursors are targeted by Sec63p has not yet been identified in mammals. However, it has been demonstrated in yeast that, Sec63p is involved in post-translational translocation of invertase, CPY and DPAP B [15,20,21]. Invertase, encoded by SUC2, is a sucrose hydrolyzing enzyme that cleaves sucrose into the glucose, and therefore plays a central role in carbohydrate metabolism [39]. Carbohydrate metabolism controls the mammalian development during the pre- and peri-implantation period [40,41], and the abnormal glucose level in uterus disrupts cell apoptosis during embryonic development[41]. CPY is a yeast vacuolar serine protease with structural homology to Cathepsin A in mammals. Cathepsin A is involved in the C-terminal processing of some implantation-related peptides/proteins including endothelin, substance P, and bradykinin [42]. Deficiency of cathepsin A leads to the lysosomal storage disease, galactosialidosis, in human [43]. DPAP B, another membrane protein of the yeast vacuole, is significantly homologous to mammalian dipeptidyl peptidase IV (DPP IV) [44]. Active DPP IV is present in glandular cells and surface epithelium during the peri-implantation period, and regarded as a differentiation marker for glandular cells; however, DPP IV enzyme activity is not detected in endometrial stromal and decidual cells throughout the menstrual cycle or during early pregnancy [45].

In addition, mutations in SEC63 leads to an autosomal dominant disease resulted from defects in protein processing, PCLD [38]. Most recently, it has been reported that, the expression of cell adhesion molecules, E-cadherin and Ep-CAM (epithelial cell adhesion molecule), were remarkably decreased in liver tissue of PCLD [46]. This suggests that, Sec63p might directly or indirectly regulate the secretion of these cell adhesion molecules. Cell adhesion molecules including integrins, selectins, and cadherins are crucial required for the successful interaction between embryo and endometrium. More interestingly, being similar to Sec63p in uterine expression pattern, a high level of E-cadherin expression was also detected specifically at the implantation sites in the endometrium of peri-implantation uterus[47]. Ep-CAM is a glycoprotein presented on most carcinomas, and has positive effects on the epithelial cell proliferation [48]. But, the expression pattern of Ep-CAM in uterus is still needed to be investigated. Based on above mentioned clues, we speculated that, during the process of deciduzlization, the intracellular expression of Sec63p is up-regulated and subsequently accelerate the decidualizing stromal cells and decidual cells to secret molecules involved in the regulation of cell growth or/and cell adhesion during the formation of deciduas.

Furthermore, in present study, a strong staining of Sec63p at the ectoplacental cone on day 8 of pregnancy was also detected. It has been well understood that, local deciduo-placental autocrine-paracrine regulation is essential for the maintenance of pregnancy, and the expression of numerous secretary proteins at the ectoplacental cone have been reported in mice. Selenoprotein P (Sepp), an extracellular glycoprotein, is a selenium transporter and antioxidant. *SEPP *mRNA was detected principally in the ectoplacental cone since embryonic day 7.5 in mice [49]. Detectable amounts of IL-18 were seen in the basal proliferative stroma in the peri-implantation period, and a high level of IL-18 expression was observed in the ectoplacental cone in the immediate post-implantation period [50]. Thus, Sec63p might play important roles not only in embryo implantation, in particular in decidualization, but also in early placentation in mice.

Collectively, we demonstrated the abundant expression of *SEC63 *gene in the mouse and human decidualized cells during early pregnancy in this study. This is consistent with the transformation of uterine stromal cells into secretory decidual cells during the process of decidualization. Furthermore, the confirmation of uterine expression of Sec63 in the human tissues further strengthens the relevance of the mouse-derived data to the situation of human pregnancy. However, further studies are required to determine the exact function of Sec63p in the uterus and the significance of its up-regulated expression at the implantation site.

## Conclusion

Taken together, our results indicated that uterine *SEC63 *gene expression is up-regulated at the implantation sites during the early pregnancy, consistent with a possible role in regulating the production of some secretory proteins during embryo implantation, especially at the stage of decidualization.

## Abbreviations

ALP: avidin-alkaline phosphatase; BCIP: 5-bromo-4-chloro-3-indolyl-phosphate; BiP: IgG heavy chain-binding protein; BSA: bovine serum albumin; CPY: carboxypeptidase Y; DIG: Digoxigenin; DPAP B: dipeptidyl-aminopeptidase B; EB: ethidium bromide; ER: endoplasmic reticulum; HRP: horseradish peroxides; IHC: immunohistochemistry; NBT: nitro-blue tetrazolium; PBS: phosphate-buffered saline; PCLD: polycystic liver disease; RT: Reverse transcription; SDS: sodium dodecyl sulfate; SRP: signal recognition particle; SSC: sodium chloride, sodium citrate.

## Competing interests

The authors declare that they have no competing interests.

## Authors' contributions

RWS carried out the *in situ *hybridization and immunohistochemical studies of mouse uterine tissues. ZGS carried out the RT-PCR and real-time quantitative PCR studies, and performed the statistical analysis. YCZ participated in the *in situ *hybridization and immunohistochemical studies of mouse uterine tissues. QJC carried out the immunohistochemical studies of human uterine and oviductal tissues. ZMY participated in the design of the study and coordination and helped to draft the manuscript. RSL participated in the design of the study and helped to draft the manuscript. JW conceived of the study and participated in its design, and drafted the manuscript. All authors read and approved the final manuscript.

## Supplementary Material

Additional file 1 **Melting curve of real-time quantitative PCR analysis for Sec63 mRNA.** The melting curves were directly exported from the qRT-PCR system, of which peaks showed the maximum melting of amplified products. The melting temperature for Sec63 was lower than that for β-actin. A single peak for each of them indicated that their corresponded primers were specific. Click here for file

## References

[B1] Aplin JD, Kimber SJ (2004). Trophoblast-uterine interactions at implantation. Reproductive Biology and Endocrinology.

[B2] Aplin JD (2006). Embryo implantation: the molecular mechanism remains elusive. Reproductive BioMedicine Online.

[B3] Paria BC, Huet-Hudson YM, Dey SK (1993). Blastocyst's state of activity determines the 'window' of implantation in the mouse receptive uterus. Proceedings National Academy of Science USA.

[B4] Makrigiannakis A, Minas V, Kalantaridou SN, Nikas G, Chrousos GP (2006). Hormonal and cytokine regulation of early implantation. Trends in Endocrinology and Metabolism.

[B5] Nie GY, Butt AR, Salamonsen LA, Findlay JK (1997). Hormal and non-hormonal agents at implantation as targes for contraception. Reproduction, Fertility and Development.

[B6] Carson DD, Bagchi I, Dey SK, Ender AC, Fazleabas AT, Lessey BA, Yoshinaga K (2000). Embryo implantation. Developmental Biology.

[B7] Achache H, Revel A (2006). Endometrial receptivity markers, the journey to successful embryo implantation. Human Reproduction Update.

[B8] Huang ZP, Ni H, Yang ZM, Wang J, Tso JK, Shen QX (2003). Expression of regulator of G-protein signaling protein 2 (RGS2) in the mouse uterus at implantation sites. Reproduction.

[B9] Sun ZG, Su RW, Yang ZM, Shi HJ, Liu CQ, Wang J (2008). Expression of the novel gene embryo implantation factor 2 (EMO2) in the mouse uterus at the implantation sites. Fertil Steril.

[B10] Willer M, Jermy AJ, Young BP, Stirling CJ (2003). Identification of novel protein-protein interactions at the cytosolic surface of the Sec63 complex in the yeast ER membrane. Yeast.

[B11] Gillece P, Pilon M, Romisch K (2000). The protein translocation channel mediates glycopeptide export across the endoplasmic reticulum membrane. Proceedings of the National Academy of Sciences of the United States of America.

[B12] Drenth JP, Martina JA, Kerkhof R van de, Bonifacino JS, Jansen JB (2005). olycystic liver disease is a disorder of cotranslational protein processing. Trends in Molecular Medicine.

[B13] Young BP, Craven RA, Reid PJ, Willer M, Stirling CJ (2001). Sec63p and Kar2p are required for the translocation of SRP-dependent precursors into the yeast endoplasmic reticulum in vivo. The EMBO Journal.

[B14] Davila S, Furu L, Gharavi AG, Tian X, Onoe T, Qian Q, Li A, Cai Y, Kamath PS, King BF, Azurmendi PJ, Tahvanainen P, Kääriäinen H, Höckerstedt K, Devuyst O, Pirson Y, Martin RS, Lifton RP, Tahvanainen E, Torres VE, Somlo S (2004). Mutations in SEC63 cause autosomal dominant polycystic liver disease. Nature Genetics.

[B15] Rothblatt JA, Deshaies RJ, Sanders SL, Daum G, Schekman R (1989). Multiple genes are required for proper insertion of secretory proteins into the endoplasmic reticulum in yeast. Journal of Cell Biology.

[B16] Sknwronek MH, Rotter M, Haas IG (1999). Molecular characterization of a novel mammalian DnaJ-like Sec63p homolog. Biological Chemistry.

[B17] Scidmore MA, Okamura HH, Rose MD (1993). Genetic interactions between KAR2 and SEC63, encoding eukaryotic homologus of DnaK and DnaJ in the endoplasmic reticulum. Molecular Biology of the Cell.

[B18] Jermy AJ, Willer M, Davis E, Wilkinson BM, Stirling CJ (2006). The Brl domain in Sec63p is required for assembly of functional endoplasmic reticulum translocons. Journal of Biological Chemistry.

[B19] Wang X, Johnsson N (2005). Protein kinase CK2 phosphorylates Sec63p to stimulate the assembly of the endoplasmic reticulum protein translocation apparatus. Journal of Cell Science.

[B20] Brodsky JL, Goeckeler J, Schekman R (1995). BiP and Sec63p are required fro both co- and posttranslational translocation into the yeast endoplasmic reticulum. Proceedings of the National Academy of Sciences of the United States of America.

[B21] Stirling CJ, Rothblatt J, Hosobuchi M, Deshaies R, Schekman R (1992). Protein translocation mutants defective in the insertion of integral membrane proteins into the ER. Mol Biol Cell.

[B22] Weitzmann A, Baldes C, Dudek J, Zimmermann R (2007). The heat shock protein 70 molecular chaperone network in the pancreatic endoplasmic reticulum – a quantitative approach. FEBS Journal.

[B23] Spencer TE, Bazer FW (2004). Uterine and placental factors regulating conceptus growth in domestic animals. J Anim Sci.

[B24] Livak KJ, Schmittgen TD (2001). Analysis of relative gene expression data using real-time quantitative PCR and the 2(-Delta Delta C(T)) Method. Methods.

[B25] Sambrook J, Fitsch EF, Maniatis T (1989). Molecular Cloning: A Laboratory Manual. Cold Spring Harbor.

[B26] Cameo P, Srisuparp S, Strakova Z, Fazleabas AT (2004). Chorionic gonadotropin and uterine dialogue in the primate. Reproductive Biology and Endocrinology.

[B27] Gellersen B, Brosens IA, Brosens JJ (2007). Decidualization of the human endometrium: mechanisms, functions, and clinical perspectives. Seminars in Reproductive Medicine.

[B28] Kennedy TG, Gillio-Meina C, Phang SH (2007). Prostaglandins and the initiation of blastocyst implantation and decidualization. Reproduction.

[B29] White CA, Robb L, Salamonsen LA (2004). Uterine extracellular matrix components are altered during defective decidualization in interleukin-11 receptor alpha deficient mice. Reproductive Biology and Endocrinology.

[B30] Matsumoto H, Sakai K, Iwashita M (2008). Insulin-like growth factor binding protein-1 induces decidualization of human endometrial stromal cells via alpha5beta1 integrin. Molecular Human Reproduction.

[B31] Lalitkumar PG, Sengupta J, Karande AA, Ghosh D (1998). Placental protein 14 in endometrium during menstrual cycle and effect of early luteal phase mifepristone administration on its expression in implantation stage endometrium in the rhesus monkey. Human Reproduction.

[B32] Salamonsen LA, Hannan NJ, Dimitriadis E (2007). Cytokines and chemokines during human embryo implantation: roles in implantation and early placentation. Seminars in Reproductive Medicine.

[B33] Beier HM, Beier-Hellwig K (1998). Molecular and cellular aspects of endometrial receptivity. Human Reproduction Update.

[B34] Zimmermann R, Muller L, Wullich B (2006). Protein transport into the endoplasmic reticulum: mechanisms and pathologies. Trends in Molecular Medicine.

[B35] Green N, Fang H, Walter P (1992). Mutants in three novel complementation groups inhibit membrane protein insertion into and soluble protein translocation across the endoplasmic reticulum membrane of Saccharomyces cerevisiae. Journal of Cell Biology.

[B36] Tuteja R (2007). Unraveling the components of protein translocation pathway in human malaria parasite Plasmodium falciparum. Archives of Biochemistry and Biophysics.

[B37] Kurihara T, Silver P (1993). Suppression of a sec63 mutation identifies a novel component of the yeast endoplasmic reticulum translocation apparatus. Molecular Biology of the Cell.

[B38] Waanders E, Croes HJ, Maass CN, Te Morsche RH, van Geffen HJ, van Krieken JH, Fransen JA, Drenth JP (2008). Cysts of PRKCSH mutated polycystic liver disease patients lack hepatocystin but express Sec63p. Histochemistry and Cell Biology.

[B39] Trumbly RJ (1992). Glucose repression in the yeast Saccharomyces cerevisiae. Molecular Microbiology.

[B40] Leese HJ (1995). Metabolic control during preimplantation mammalian development. Human Reproduction Update.

[B41] Pampfer S (2000). Peri-implantation embryopathy induced by maternal diabetes. Journal of Reproduction and Fertility Supplement.

[B42] Skidgel RA, Erdös EG (1998). Cellular carboxypeptidases. Immunological Reviews.

[B43] Kleijer WJ, Geilen GC, Janse HC, van Diggelen OP, Zhou XY, Galjart NJ, Galjaard H, d'Azzo A (1996). Cathepsin A deficiency in galactosialidosis: studies of patients and carriers in 16 families. Pediatric Research.

[B44] Marguet D, Bernard AM, Vivier I, Darmoul D, Naquet P, Pierres M (1992). cDNA cloning for mouse thymocyte-activating molecule. A multifunctional ecto-dipeptidyl peptidase IV (CD26) included in a subgroup of serine proteases. Journal of Biological Chemistry.

[B45] Imai K, Maeda M, Fujiwara H, Kariya M, Takakura K, Kanzaki H, Mori T (1992). Dipeptidyl peptidase IV as a differentiation marker of the human endometrial glandular cells. Human Reproduction.

[B46] Waanders E, Van Krieken JH, Lameris AL, Drenth JP (2008). Disrupted cell adhesion but not proliferation mediates cyst formation in polycystic liver disease. Modern Pathology.

[B47] Jha RK, Titus S, Saxena D, Kumar PG, Laloraya M (2006). Profiling of E-cadherin, beta-catenin and Ca(2+) in embryo-uterine interactions at implantation. FEBS Letters.

[B48] Balzar M, Winter MJ, de Boer CJ, Litvinov SV (1999). The biology of the 17-1A antigen (Ep-CAM). Journal of Molecular Medicine.

[B49] Lee SR, Yon JM, Baek IJ, Kim MR, Park CG, Lee BJ, Yun YW, Nam SY (2008). Spatiotemporal expression of the selenoprotein P gene in postimplantational mouse embryos. International Journal of Developmental Biology.

[B50] Ostojic S, Dubanchet S, Chaouat G, Abdelkarim M, Truyens C, Capron F (2003). Demonstration of the presence of IL-16, IL-17 and IL-18 at the murine fetomaternal interface during murine pregnancy. Am J Reprod Immunol.

