# Supplementary figures and images for: The uterine expression of SEC63 gene is up-regulated at implantation sites in association with the decidualization during the early pregnancy in mice
Source: Reprod Biol Endocrinol. 2009 Feb 11;7:12. doi: 10.1186/1477-7827-7-12 (PMC2655295; doi:10.1186/1477-7827-7-12)

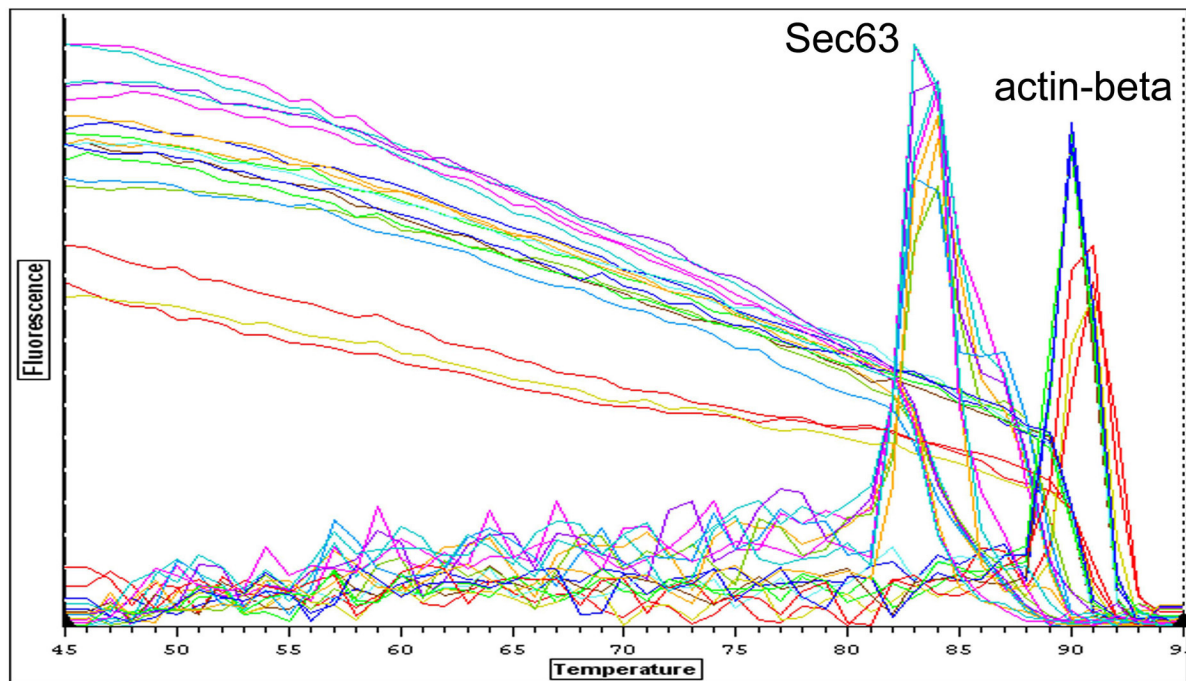

Melting Curve from 45-95°C, read every 1.0 °C, hold 10sec

Supplement: Additional file 1 — Melting curve of real-time quantitative PCR analysis for Sec63 mRNA. The melting curves were directly exported from the qRT-PCR system, of which peaks showed the maximum melting of amplified products. The melting temperature for Sec63 was lower than that for β-actin. A single peak for each of them indicated that their corresponded primers were specific. [file 1477-7827-7-12-S1.pdf]
